# Heavy Metal Bioaccumulation in European Eels (*Anguilla anguilla*) from the Odra and Vistula River Basins (Poland): Implications for Environmental and Food Safety

**DOI:** 10.3390/ani16020287

**Published:** 2026-01-16

**Authors:** Joanna Nowosad, Tomasz K. Czarkowski, Andrzej Kapusta, Natalia Mariańska, Piotr Chmieliński, Bartosz Czarnecki, Jakub Pyka, Michał K. Łuczyński, Gulmira Ablaisanova, Dariusz Kucharczyk

**Affiliations:** 1 Department of Ichthyology, Hydrobiology and Aquatic Ecology, National Inland Fisheries Research Institute, Al. Oczapowskiego 10, 10-719 Olsztyn, Poland; 2 Department of Research and Development, ChemProf, Gutkowo 54B, 11-041 Olsztyn, Poland; 3 Department of Ichthyology and Aquaculture, University of Warmia and Mazury in Olsztyn, Al. Warszawska 117A, 10-957 Olsztyn, Poland; 4 LLP Fisheries Research and Production Center, Almaty 050016, Kazakhstan

**Keywords:** aquatic environment, contamination, critically endangered species, lead, mercury, pollution

## Abstract

The accumulation of heavy metals in fish tissues is widely recognized as an indicator of aquatic environmental pollution, and the analysis of their content provides a basis for assessing ecological risk and the safety of aquatic food. The European eel (*Anguilla anguilla*) is a species frequently used as a bioindicator in environmental studies due to its wide geographic distribution, long life cycle, and high capacity for bioaccumulation of heavy metals in various tissues. The aim of this study was to assess the variation in the accumulation of heavy metals, i.e., mercury (Hg), lead (Pb), arsenic (As), and cadmium (Cd), in the tissues (muscle, liver, gonads, and gills) of European eels caught in two locations in Polish inland waters. The obtained results showed significant differences both in the concentration levels of individual elements and in their co-occurrence in the examined tissues. The statistical methods used, including correlation analysis, heat maps, and principal component analysis (PCA), allowed for a comprehensive assessment of the relationships between metals and the identification of factors differentiating the studied populations. The obtained results clearly indicate that fish residing in similar environments for long periods exhibit significant differences in heavy metal content in various fish tissues. Fish obtained from environments with potentially higher levels of heavy metal inputs, such as the Oder River EMU compared with the Vistula River EMU, showed higher levels of heavy metal accumulation in tissues. This study also found that the concentration of heavy metals tested did not exceed the safe standards for human fish consumption.

## Simple Summary

Fish living in polluted waters can absorb harmful heavy metals, which may later be passed on to people through food. For this reason, checking the levels of heavy metals in edible fish is important for both environmental protection and human health. The European eel (*Anguilla anguilla*) is especially suitable for such studies because it has a long lifespan and easily accumulates pollutants in its body. In this study, the content of mercury, lead, arsenic, and cadmium in different tissues (muscles, liver, gonads, gills) of European eels collected from two inland water bodies in Poland was examined. Clear differences in metal levels were observed between organs and between the two locations. It was found that the concentration of the tested heavy metals did not exceed the safe limits for human consumption of fish. These results show that the European eel can help assess water pollution and support the evaluation of potential health risks related to fish consumption.

## 1. Introduction

In recent decades, pollution of the aquatic environment with heavy metals has become one of the key ecological problems, significantly affecting the structure and functioning of ecosystems, the health of aquatic organisms, and the safety of fish consumers [1,2,3]. Metals such as mercury (Hg), cadmium (Cd), arsenic (As), and lead (Pb) are particularly dangerous due to their high toxicity, persistence in the environment, and ability to bioaccumulate in aquatic organisms [4]. The bioaccumulation of heavy metals in aquatic organisms, including fish, is a process involving the accumulation of toxic elements in their tissues because of contact with contaminated aquatic environments, bottom sediments, and contaminated food [5]. Heavy metals are characterized by chemical forms that are resistant to degradation and tend to accumulate in organisms at higher trophic levels. Aquatic animals, especially those at the top of the trophic pyramid, are particularly vulnerable to heavy metal pollution, which can lead to ecological imbalances, e.g., reproductive impairment [6,7,8]. As a result, this situation can weaken and disrupt the food and trophic chains [5]. In fish, the bioaccumulation of Hg, Cd, Pb, and As occurs primarily in the liver and muscles, as well as the gills and gonads. The liver plays a key role in body detoxification, and the muscles constitute the main reservoir of heavy metals, which is particularly important for fish consumers [9,10,11,12]. This process is particularly intense in species with a long life cycle in a given environment with a high trophic level, such as the European eel (*Anguilla anguilla*), which makes it a valuable bioindicator of aquatic environmental pollution [13,14], not only territorially but also over time. Fish, especially long-lived ones, such as the European eel, due to their ability to metabolize, detoxify, and accumulate heavy metals in their bodies, are considered important bioindicators for monitoring the pollution of aquatic ecosystems [15], while the European eel itself is a critically endangered species and is included in the IUCN Red List of Threatened Species [16].

The literature indicates that the European eel can act as an indicator organism in assessing the quality of the aquatic environment. The European eel has a high lipid content and a benthic lifestyle, which predisposes it to the bioaccumulation of lipophilic pollutants [17,18]. Furthermore, the widespread occurrence of eels (Europe, Asia Minor, Northwest Africa) allows for comparisons between different regions and river systems regarding environmental pollutants. Studies conducted in other parts of the continent indicate significant differences in the level of metal accumulation, depending on environmental conditions, the age of the fish, and local emission sources [18]. In the case of eel, which is still an important consumer species in some European countries, this risk is particularly significant [11,19]. At the same time, this situation poses challenges for the implementation of sustainable fisheries policy and the protection of water resources within the framework of EU strategies, such as the Water Framework Directive (2000/60/EC). However, previous analyses for the Vistula and Oder River basins are few, fragmentary, and often based on data from a dozen or so years ago, which makes it difficult to assess the current ecological situation [20,21]. Many studies have shown that the level of metal bioaccumulation in fish depends on their ecological specificity, environmental conditions, and the characteristics of the metals themselves, such as their solubility, chemical form, and affinity for proteins [22,23]. Bioaccumulation also depends on factors such as age, body weight, fish size, and the level of environmental pollution [24]. The effects of heavy metal bioaccumulation are significant not only for the health of aquatic organisms themselves, but also for ecosystems and human health. Heavy metal contamination can lead to physiological disorders in fish, such as damage to the nervous system, impaired reproductive function, and reduced immunity [25]. Fish constitute a crucial link in the human food chain, and their consumption can lead to exposure to toxic elements, which necessitates the regular monitoring of heavy metal levels in the aquatic environment and fish tissues [26].

The natural habitats of *A. anguilla* in freshwater in Poland include rivers, lakes, transitional waters, and coastal marine waters with very low salinity. Poland’s territory is divided into two eel management units (EMUs): the Oder and Vistula EMUs. These eel management units vary regarding the degree of environmental transformation. The Oder EMU is significantly affected by environmental transformations, including industrial and agricultural pollution. The Oder River basin is home to Poland’s largest industrial area, and the largest ecological disaster occurred in this area [27]. The Vistula EMU, on the other hand, encompasses areas with a significant degree of naturalness, and the main habitats of *A. anguilla* are inland waters with less anthropopressure. Differences in river basin characteristics, including the intensity of human pressure and pollution sources, may influence the level of heavy metal bioaccumulation in aquatic organisms, including the European eel [28]. Eels harvested from the Vistula and Oder River basins are an important research subject due to their economic and ecological importance and as bioindicators of water quality [29,30]. At the same time, this species is particularly vulnerable to the accumulation of toxic substances in tissues such as the muscles, liver, gills, and gonads, which is important both for its health and for the quality of fish intended for human consumption. Research on the accumulation of heavy metals in the European eel in the Vistula and Oder River basins is important from both the ecological and fisheries perspectives. Assessing the level of bioaccumulation in this species enables the identification of areas of increased ecological risk, determining the potential threat to fish consumers, and providing data supporting the implementation of aquatic environment protection policies and eel population recovery programs in Central Europe [8,31].

The aim of this study was to assess the levels of heavy metals (Hg, Pb, Cd, and As) in the tissues of European eels from the Oder and Vistula EMUs, considering differences in the content of these elements between the tissues and between the two main Polish EMUs analyzed. The results allow not only to determine the degree of aquatic environmental pollution in these two areas but also to assess the potential health risks to consumers consuming eels from these regions. This study fits into the broader context of aquatic environment monitoring and food safety assessment in the context of heavy metal contamination.

## 2. Materials and Methods

### 2.1. The Origin of the Fish

The eels were collected using fyke nets. In the Vistula EMU, eels (*n* = 20) were caught in September 2024 in Lake Żarnowieckie (54.79° N, 18.05° E). In the Oder EMU, eels (*n* = 20) came from commercial catches conducted in the Oder River (53.15° N, 14.39° E) in October 2024. After being caught, the fish were frozen and transported to the laboratory, where they were then preserved for further analysis. At the laboratory of the Department of Ichthyology, Hydrobiology and Water Ecology (NIFRI, Olsztyn; Poland), all eels were assigned a unique identification number, enabling the precise tracking of biological material throughout the work.

### 2.2. Measurements and Sampling

Before sampling for analysis, each fish was weighed (±0.01 g; Radwag PS R1; Radwag, Radom, Poland) and measured (±1.0 mm). Otoliths and tissues (a fragment of muscle near the head, a fragment of the gonad, liver, and gills) were then dissected and preserved for further analysis. The sample tissues were stored in an ultra-low-temperature freezer (Sanyo, MDF-U32V; Moriguchi, Japan) at −80 °C until analysis. From the obtained data, the following parameters were calculated [32]: Fulton’s condition index (K = BW × TL^−3^; where BW: the body weight in g; TL: the fish length in cm) and hepatosomatic index (HSI = LW/BW × 100; where LW: the liver weight in g; BW: the body weight in g).

### 2.3. Determination of Fish Age

Fish age was estimated based on otolith analysis according to the methodology described by Stevenson and Campana [33] and Carbonara and Folles [34]. One of the three otoliths, the sagittal (*Sthatolithus sagitto*), was used for this purpose. After dissecting the braincase, the otoliths were cleaned of tissue debris and embedded in synthetic resin (Entelan; Sigma-Aldrich; Darmstadt, Germany). The embedded otoliths were polished with progressively gritted paper (P800, P1200, and P1500). To stain the rings embedded in the otoliths, they were stained with toluidine blue (Kolchem; Łódź, Poland). After drying, the stained surface was coated with a thin, transparent layer of synthetic resin (Entelan; Sigma-Aldrich; Darmstadt, Germany) to preserve the specimen and protect it from adverse conditions. The closed, dark blue rings extending outward at various distances from the otolith center represented the age. Each otolith was photographed using an Optika camera (OptikaKam PRO 3 Digital Camera; OPTIKA S.r.l., Ponteranica, Italy) mounted on a Nikon magnifying glass (SMZ 745T; Tokyo, Japan). Age was estimated from the photographs [35].

### 2.4. Analysis of Swim Bladder Nematode Infection

The analysis of the swim bladder infection with the nematode *Anguillicoloides crassus* was performed on all captured European eels. After determining the weight and total body length, the body cavity was opened along the ventral line. The swim bladder was cut lengthwise. All nematodes visible to the naked eye in the lumen of the swim bladder and in the airway were identified.

### 2.5. Sample Mineralization

Fish tissues (liver, gonads, gills, muscles) were weighed on a Radwag precision balance (Poland) to the nearest 0.001 g. The samples were placed in Teflon vessels (PerkinElmer; Waltham, MA, USA) and filled with 10 mL of nitric acid (69–70%; Baker Instra-Analyzed Reagent, Phillipsburg, NJ, USA). After tightly closing the vessels, they were placed in a mineralizer (Titan MPS; PerkinElmer, Waltham, MA, USA) and subjected to a microwave-assisted pressure digestion process. The contents of the vessels were poured into flasks and made up to 25 mL with ultrapure water [36,37].

### 2.6. Determination of Heavy Metal Levels

The elemental content of the samples was analyzed using inductively coupled plasma mass spectrometry (ICP-MS) (NexION 2000, Perkin Elmer, Waltham, MA, USA). This technique allowed for the precise determination of trace elements in food, pharmaceutical, and environmental samples. ICP-MS is particularly useful for determining the low concentrations of heavy metals such as mercury (Hg), cadmium (Cd), lead (Pb), and arsenic (Ar). Mass spectrometry allows for the determination of elemental concentrations at very low detection limits, enabling precise analyses even for trace amounts of these elements. The detection limits for mercury, cadmium, lead, and arsenic were 0.005, 0.002, 0.010, and 0.006 ppm, respectively. Such low detection limits make the ICP-MS method the preferred method for the analysis of samples that must meet strict safety standards in the context of food regulations or environmental monitoring [38,39]. Best practices were followed for the quality control of the analytical determination procedures such as the ICP-MS analysis. Several quality control measures were implemented to ensure the reliability and accuracy of the ICP-MS analytical determinations. Calibration curves were established using certified multi-element standard solutions (ICP Multi element standard solution IX; LOT No.: 1008987; CPAchem, Bogomilovo, Bulgaria). Calibration linearity was verified (R^2^ > 0.999 for all analyzed elements). Procedural blanks were included in each batch of analyses to check for impurities and background signals. Analytical accuracy was verified by analyzing the appropriate certified reference materials under the same conditions as the samples. All samples were prepared at least as duplicates and measured at least three times. To assess the precision, relative standard deviations (RSDs) were calculated, with all determinations being below 5%.

### 2.7. Statistical Analysis

The results are presented as the mean with standard error (mean ± SE). Statistica 13.1 analysis was performed (TIBCO Software Inc.; Palo Alto, CA, USA). To demonstrate differences between two groups (e.g., fish age, heavy metal concentrations in the same tissue in fish from two locations), Student’s *t*-test was used, assuming a significance level of α = 0.05 (*p* < 0.05). One-way ANOVA and Tukey’s test were used to demonstrate statistically significant differences for the comparison of more than two groups (i.e., the comparison of heavy metal content in different tissues). Fisher’s exact test was used to compare the frequency of parasites between fish from the Oder and Vistula River basins. Spearman’s correlation was used to determine the relationship between mercury concentration in muscle and fish age. Heavy metal correlation heat maps for eels from the Vistula and Odra rivers were generated in Python v3.11 (Python Software Foundation (PSF); Beaverton, OR, USA) using the Seaborn library (v0.13.2). Pearson correlation coefficients for heavy metals (Hg, Cd, Pb, and As) in different tissues are presented as color matrices. Principal component analysis (PCA) was performed and the graph was generated using Statistica 13.1.

## 3. Results

### 3.1. Fish

This study of heavy metals (mercury, cadmium, lead, and arsenic) was conducted using 20 eel individuals caught in the Vistula River basin and 20 individuals caught in the Oder River basin. The mean weight of fish from the Vistula River basin was 265.30 ± 13.94 (184–382) g and from the Odra River basin was 428.53 ± 47.29 (158–932) g (Table 1). The mean age of fish differed statistically significantly between the fish populations caught from the Vistula and Oder River basins (Student’s *t*-test showed a significant difference; *p* = 0.00003). It was found that more than 70% of the individuals were infected with *Anguillicoloides crassus* (70% and 77% from the Odra and Vistula River basins, respectively), but the analysis did not show significant differences between the groups (Fisher’s test, *p* = 0.72).

### 3.2. Mercury Concentration (Hg)

Analysis of the obtained results revealed significant differences in heavy metal concentrations in various tissues of European eel from the Vistula and Oder River basin EMUs. The results indicate significant variation in the content of mercury, cadmium, lead, and arsenic, although not all these differences were statistically significant.

**Figure 1:**
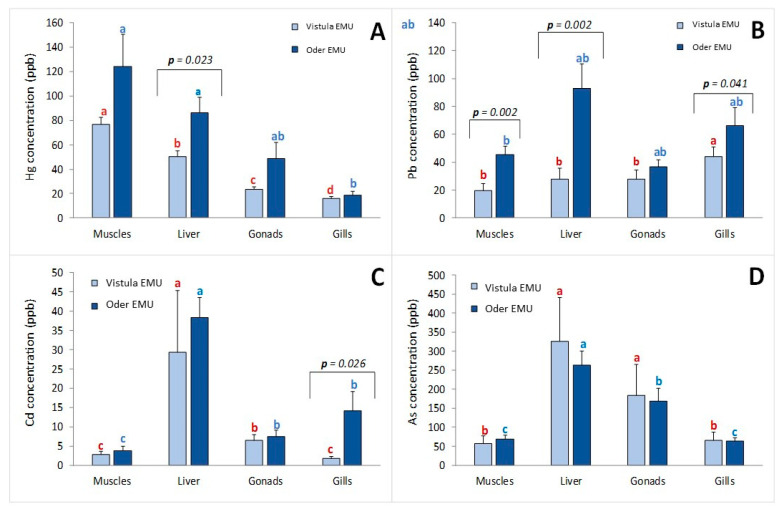
Heavy metal content (ppb; mean ± SE) of (**A**) mercury (Hg), (**B**) lead (Pb), (**C**) cadmium (Cd), and (**D**) arsenic (As) in the gills, liver, muscles, and gonads of European eels (*Anguilla anguilla*) caught in the Vistula and Odra Rivers basins (EMUs). Data marked with different letter indices of the same color indicate statistically significant differences (Tukey test; *p* < 0.05). Data marked with a bracket indicate statistically significant differences for the same tissue from fish from different environments (*t*-test; *p* < 0.05).

Analysis of heavy metal content in the gills of European eel showed varying heavy metal concentrations depending on the location of fish capture (Figure 1A). The highest mercury concentrations were recorded in the muscles of fish from the Odra River, in which the mean concentration was 124.06 ± 26.48 (28.89–482.72) ppb, exceeding the levels found in the analogous samples from the Vistula River basins (76.61 ± 6.09 (31.65–137.79) ppb). These differences were not significant due to large differences in Hg levels in fish from the Odra River (*p* > 0.05). A significantly higher mercury concentration was found in the liver of the fish caught from the Odra River basin (85.99 ± 12.69 ppb) compared with the fish from the Vistula River basin (50.12 ± 4.72 ppb) (*t*-test; *p* = 0.023). In contrast, in the gonads and gills, the differences in Hg concentration between the rivers were less pronounced. The Hg accumulation profile in both populations can be presented as follows: muscles > liver > gonads > gills (Figure 1A).

### 3.3. Lead Concentration (Pb)

The lead content was significantly higher in the tissues of fish from the Odra River compared with those from the Vistula River (*p* = 0.002 for muscles; *p* = 0.002 for liver; *p* = 0.0407 for gills). The highest Pb concentrations in fish caught in the Vistula River basin were found in the gills (43.80 ± 7.11 ppb) and in the liver (92.94 ± 17.32 ppb) in fish from the Odra River basin. The Pb level in the liver of fish from the Odra River basin was more than twice as high as in fish from the Vistula River basin (27.86 ± 7.99 ppb). No significant differences were found between Pb concentrations in muscles, liver, and gonads in fish caught in the Vistula River basin (Figure 1B).

### 3.4. Cadmium Concentration (Cd)

The liver was the most heavily burdened with cadmium, particularly in fish from the Odra River basin, where the mean Cd concentration was 38.36 ± 5.18 ppb, while in fish from the Vistula River basin, these values were lower at 29.37 ± 15.99 ppb. Analyzing cadmium (Cd) concentrations, it was noted that there was significantly less Cd in the muscle tissue than in the gonads of the fish from the Vistula and Odra River basins (*p* < 0.05; Figure 1C). A significantly higher Cd concentration was found in the gills of fish from the Odra vs. the Vistula River (*p* = 0.026).

### 3.5. Arsenic Concentration (As)

The order of As distribution in fish tissues is as follows: liver > gonads > muscles > gills. Arsenic was present in the highest concentrations in the liver and gonads, particularly in individuals from the Odra River (Figure 1D). The As concentrations in the liver were 326.72 ± 114.52 ppb in the Vistula EMU and 263.39 ± 37.62 ppb in the Oder EMU. In turn, the arsenic (As) concentrations in the gills were at a moderate level and amounted to 63.80 ± 7.82 ppb in the Oder EMU and 64.62 ± 22.66 ppb in the Vistula EMU.

**Figure 2:**
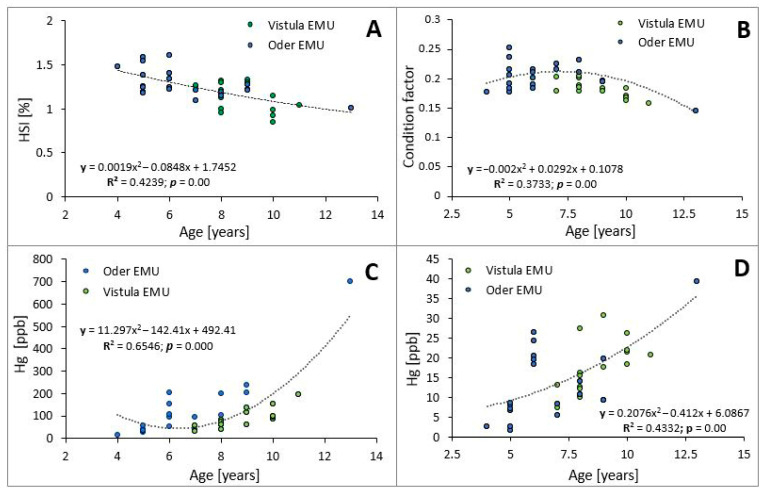
The relationship between the age and the hepatosomatic index (HSI) (**A**), Fulton’s condition index (**B**), and mercury content in the muscle tissue (**C**) and gills (**D**) of European eels (*Anguilla anguilla*; *n* = 40), caught in the Vistula and Oder River basins.

Analysis of the relationship between the age of European eels and the HSI value (R^2^ = 0.4239; *p* = 0.000; Figure 2A) and Fulton’s condition index (R^2^ = 0.3733; *p* = 0.000; Figure 2A) showed a significant negative correlation. With age, a lower condition index value and a lower relative liver weight in relation to body weight were observed. Furthermore, a significant positive correlation was observed between mercury content in muscles (R^2^ = 0.6546; *p* = 0.000) and gills (R^2^ = 0.4332; *p* = 0.000) of fish and their age (Figure 2C,D). With age and lifespan, higher mercury concentrations were found in muscles and gills.

**Figure 3:**
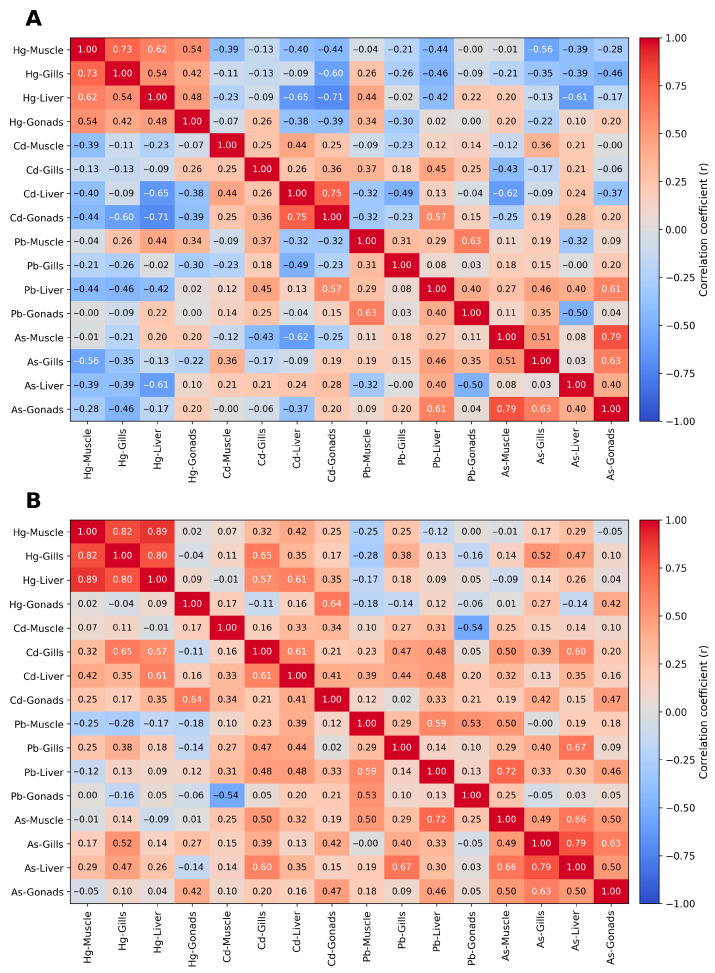
Heat map correlation of heavy metal content (Hg: mercury; Cd: cadmium; Pb: lead; As: arsenic) in tissues of European eel (*Anguilla anguilla*) from Vistula (**A**) and Oder (**B**) River basins (EMUs), Poland.

Data visualization using heat maps (Figure 3) revealed strong positive correlations between mercury concentrations in the muscles, gills, and liver (r = 0.65–0.76; Figure 3) and cadmium concentrations in the muscles and gills (r = 0.82) and a correlation of lead with arsenic in the gonad and muscle tissues (r = 0.55–0.68). In the case of mercury and cadmium accumulation in tissues, negative correlations (up to −0.5) were observed in fish from the Vistula River basins. In the case of fish from the Oder River (Figure 3B), stronger tissue correlations with heavy metals were observed (r = 0.86–0.89 for Hg-muscle, Hg-gills, and Hg-liver). Moreover, positive correlations were also found between Cd-gills and Cd-liver, Cd-muscle, and Cd-liver (r = 0.34–0.75), and for As and Pb, numerous positive correlations (r = 0.55–0.73) were observed, especially in the liver and gills (Figure 3B).

**Figure 4:**
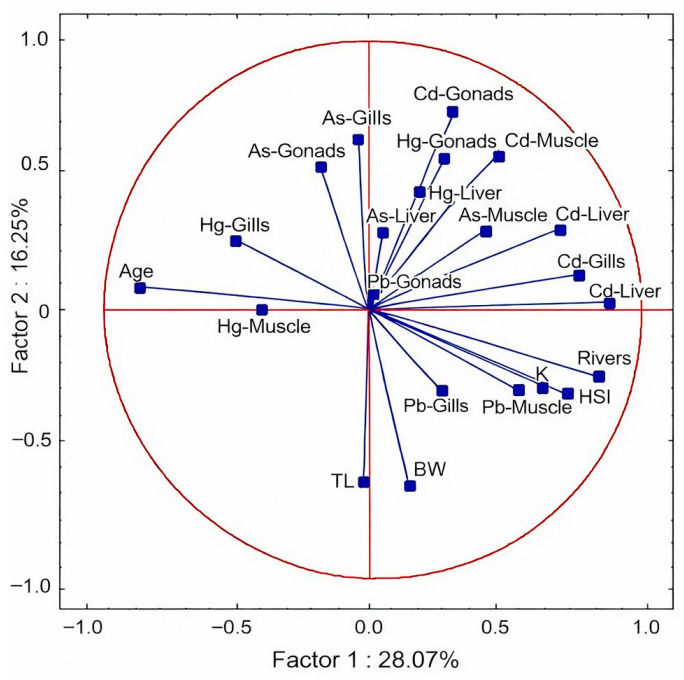
Biplot principal component analysis (PCA) showing correlations between heavy metal (Hg, Cd, Pb, As) concentrations in various tissues (muscles, liver, gonads, gills) of European eel (*Anguilla anguilla*) from the Vistula and Oder River basins (EMUs). The analysis includes fish codification data such as LT (body length), BW (body weight), K (Fulton’s condition index), and HSI (hepatosomatic index). Red circle: confidence limit (95%) identification of outlier samples; Red lines: principal component axes (PC1 and PC2), center of the coordinate system; Blue vectors: variable loadings, show how strongly and in what direction individual variables influence the main components of PCA.

Principal component analysis performed for European eel samples showed that the first two components together explain 44.3% of the total variability in the data. The highest positive loadings were obtained for variables related to the content of Cd, Pb, and As in tissues (Factor 1), while morphometric variables, such as total length (TL) and body weight (BW), were located on the negative side. The second component (Factor 2) mainly explains the differences between Hg levels and Cd and As levels, indicating a different nature of mercury accumulation compared with other metals (Figure 4).

## 4. Discussion

Water pollution due to heavy metals is a significant environmental problem, affecting both the stability of ecosystems and the health of aquatic organisms [40]. Surface and groundwater worldwide are increasingly exposed to chemical pollutants, particularly heavy metals, which originate from both natural sources, such as rock weathering, and anthropogenic sources, such as industry, agriculture, municipal sewage, and industrial waste. Pollution of surface and groundwater with heavy metals, such as mercury (Hg), cadmium (Cd), arsenic (As), and lead (Pb), is becoming a growing environmental and health problem worldwide [41,42,43]. Heavy metals are persistent and can bioaccumulate in aquatic organisms, especially in fish, and their consumption contributes to physiological and neurological disorders and cancer risk in humans and animals [11,12,44,45]. Many scientific reports indicate that average concentrations of Pb and Hg in surface waters exceed the permissible limits of the World Health Organization (WHO) in all continents, while Cd and As also often occur at elevated levels, especially in Asia, Africa, and South America [5,42,46,47,48,49,50]. Local geographical differences, climate, catchment structure, land use, and anthropogenic sources significantly influence the pollution of the aquatic environment [51]. Studies on the distribution of heavy metals in the aquatic environment worldwide as well as in Poland (this study) show that pollution levels may differ significantly even between rivers located at the same latitude [5,52,53]. Numerous studies show that the degree of water pollution is reflected in the content of heavy metals in fish tissues [5,49,50].

The European eel is particularly susceptible to heavy metal accumulation in its tissues due to its long life cycle, intercontinental migrations between various aquatic ecosystems, and contact with benthos, which is one of the main reservoirs of environmental pollutants, including heavy metals in the aquatic environment [25]. Therefore, several studies report that the heavy metal content in European eel tissues constitutes an important indicator for assessing the quality of the aquatic environment and the potential risk to fish consumers. This study analyzed the heavy metal content (As, Cd, Hg, and Pb) in the tissues of European eels caught in the Vistula and Oder River basins. Furthermore, the relationship between the age of European eels and the Fulton’s condition index (K) and HSI was analyzed. Eels, due to their long lifespan and specific mode of existence in bottom sediments, are particularly vulnerable to the accumulation of toxic elements [54,55]. Numerous studies indicate that the age of fish is an important factor for determining the level of heavy metal accumulation, i.e., older individuals usually show higher concentrations due to longer exposure time [56], which was also confirmed in this study. The Oder River flows through the highly industrialized region of Upper Silesia and has a transboundary catchment (Czech Republic, Germany) [57]. Long-term mining and industry have increased the load of salts and metals in the Oder system, which was noted, among others, in sediments and floodplain soils [58]. In 2022, it experienced an ecological disaster related to a mass fish kill caused by a bloom of the toxic alga *Prymnesium parvum*, which developed under high salinity conditions caused by industrial discharges [59]. The relatively low heavy metal content found in the tissues of European eel from Lake Żarnowieckie may be largely related to the hydrological conditions and management of its catchment area. The lake’s catchment area primarily consists of agricultural and forest areas, with a small proportion of urbanized areas that lack high-risk industries. The lake’s main tributary is the Piaśnica River, which is characterized by good water chemical status. Numerous studies indicate that intensive urbanization and industrial development in lake catchments lead to the increased transport of heavy metals, which is reflected in their concentrations in sediments and fish tissues, whereas in agricultural and forest catchments, metal loads are generally lower [60]. In this study, eels from Lake Żarnowieckie in the Vistula River basin are relatively less burdened with heavy metals than their populations from highly urbanized and industrialized catchments. Consequently, the relatively low concentrations of heavy metals in eel tissues from Lake Żarnowieckie may reflect both the favorable hydrological conditions of the reservoir and limited anthropogenic pressure in its catchment. These results confirm that local environmental conditions play a key role in shaping the level of metal bioaccumulation in fish organisms.

Differences in the level and structure of metal accumulation may result from different physicochemical conditions of river waters, such as pH, organic matter content, water hardness, or the nature of bottom sediments. Age and condition of fish may also have a significant impact—older individuals, especially from the Oder River, could accumulate Hg over a longer period, as also noted in the study by Pierron et al. [54]. Condition factors (K) and hepatosomatic indices (HSIs) indicated the good overall condition of the studied individuals, suggesting that the observed differences are likely environmental in nature, not physiological. This may also be evidenced by the fact that, in the eels in this study, a significant positive correlation (*p* < 0.05) was found between heavy metal content in the muscles and gills. Under the conditions of heavy metal contamination, the gills, as an organ in direct contact with water, usually show higher metal levels than the muscles [61,62]. The relationship between the metal levels in the muscles and gills may be variable and dependent on the species and habitat, as reported by Hlatshwayo et al. [63].

Analysis of the results obtained in this study suggests that the fish from the Oder River basin were characterized by higher levels of the analyzed heavy metals (Hg, Pb, Cd, and As) in most of the examined tissues compared with the fish from the Vistula River basin. The liver consistently showed the highest degree of heavy metal accumulation, confirming its function as a detoxification organ. The comparison of results between eel populations from the Vistula and Oder River basins revealed distinct differences in the structure of contaminants. Fish from the Oder River showed greater divergence in metal profiles, which may indicate more widespread sources of contamination within this river basin. In contrast, fish from the Vistula River basin showed greater individual variability, particularly in the Cd and As concentrations, which may be related to the more developed nature of the basin, encompassing both agricultural areas and areas with limited anthropogenic impact. Similar relationships were described by Couture and Rajotte [64] and Havelkova et al. [52], indicating that in fish populations from rivers with a high share of heavy industries, metal accumulation profiles are similar. The correlation heat map (Figure 3) showed strong positive relationships between Hg, Pb, and As content, especially in samples from the Oder River, suggesting common sources of industrial pollutant emissions and similar accumulation mechanisms. In samples from the Vistula River basin, these relationships were weaker and less regular, which may indicate greater diversity of local pollution sources. High correlations between metals with similar chemical properties, such as Cd and Pb, also confirm their cooperation in the processes of transport into the body and accumulation in tissues. In turn, the negative correlations between Hg and Cd observed in some tissues may result from competition for binding sites with muscle proteins, as also noted by Pierron et al. [54].

PCA confirmed that the variability in heavy metal accumulation is largely dependent on the habitat. The first principal component, explaining 26.4% of the total variance, was strongly related to mercury (Hg) content in the muscles and liver, while the second component reflected a variation in cadmium (Cd) and lead (Pb) content in metabolic tissues. Therefore, the principal component analysis in this study reflects the different pathways of heavy metal uptake and metabolism in the European eel. These results, indicating diverse patterns of heavy metal accumulation in European eel tissues, are consistent with observations made by other authors. Similarly to the studies by Polak-Juszczak [65], Pierron et al. [54], Nowosad et al. [7,8,66], and Polak-Juszczak and Szlider-Richert [67], the highest concentrations of mercury (Hg) were recorded in the muscles, confirming its strong trophic bioaccumulation and association with fish weight. In turn, Cd and Pb in PCA clustered near organ variables (liver, gills), showing correlations similar to those described by Batty et al. [46], Linde et al. [68], and Zaghloul et al. [69] for the European eel. The apparent separation of variables suggests that heavy metals group according to the site of their highest accumulation in the body (e.g., Cd and Pb in the liver and gills, Hg in the muscles), which may be related to different pathways of their uptake and metabolism in the European eel. Pyle et al. [70] suggested that Ca ion uptake through the gill’s limits Cd absorption from food in environments with high water hardness. However, high arsenic (As) concentrations in the liver and gills observed in this study are also consistent with the results of Tolkou et al. [71], indicating the dominant accumulation of this element in tissues with intensive metabolism and direct contact with water. High arsenic (As) values in the liver and gills observed in this study indicate that these organs are the main sites of accumulation of this element in fish inhabiting river environments with increased anthropogenic impact [71]. Differences in the direction and strength of the vectors in the PCA plot (Figure 3) may therefore reflect the differential bioavailability of elements and local environmental conditions in the studied rivers.

Heavy metals can be hazardous to the health and functioning of various groups of organisms and can even be a direct cause of death in humans [72,73,74]. The muscles, as a tissue important for human consumption, exhibited significant accumulation of Hg and Pb, which may pose a potential health risk to consumers. However, this study demonstrated that the concentrations of heavy metals such as mercury, cadmium, and lead in the European eel meat are within the acceptable regulatory limits, indicating no direct health risk to consumers. Statistical differences between fish from the Vistula and Oder Rivers indicate possible differences in environmental pollution levels between these two river basins. Regarding human consumption, the concentrations of inorganic arsenic, cadmium, mercury, and lead determined in the European eel (*Anguilla anguilla*) muscle tissue were below the maximum levels established in Commission Regulation (EU) 2023/915, as amended, and therefore do not indicate a health risk to consumers [75].

Although studies have shown that the levels of heavy metals in the meat of the tested fish are within acceptable consumer limits, it is important to remember that even low concentrations of metals can negatively impact fish reproductive processes. Fish exposure to heavy metals can lead to disruptions in their reproductive capacity, which can have serious consequences for the number and health of their offspring. Many studies report that heavy metals lower the gonadosomatic index, delay oogenesis, impair the quality of gametes, and reduce the survival of embryos and larvae, which can lead to a long-term decline in the reproductive success of the population [6,76]. In the case of the European eel, contamination with heavy metals and other xenobiotics is considered one of the significant factors impairing the quality of spawners and, thus, the reproductive potential of this species [6,77]. Studies have shown the accumulation of cadmium in the muscles, ovaries, and eggs of female eels, as well as the transfer of this metal to oocytes, poses a risk of impaired development of embryos and offspring [8]. At the same time, as confirmed in this study, the numerous populations of European eels are infected with the nematode *Anguillicoides crassus*, which damages the swim bladder, reduces swimming capacity, and significantly impedes the long-distance spawning migration to the Sargasso Sea [78,79,80,81]. Toxicological stress from heavy metals and parasitism can significantly limit the ability of some individuals to swim to spawning grounds and reduce the reproductive success of the entire population [77,80,81]. Therefore, monitoring the concentration of heavy metals in aquatic environments and fish tissues is crucial for protecting biodiversity and ensuring population stability.

## 5. Conclusions

The study results showed that the European eel population from the Oder River basin is characterized by higher and more variable levels of heavy metal accumulation compared with the population from the Vistula River basin. However, the fish from the Vistula River basin exhibited high variability in cadmium and arsenic concentrations in the liver, which may suggest the occurrence of point source pollution for these elements. Metabolic tissues (liver, gills) were the main sites of Cd and Pb accumulation, while Hg accumulated primarily in the muscles. Multivariate analyses confirmed the existence of significant environmental differences between the studied river basins, resulting from different pollution sources and the nature of the catchment areas. Despite this, the tested fish did not exhibit hazardous levels of heavy metals (As, Cd, Hg, Pb) in the muscle tissue and can therefore be considered safe for consumption.

## Figures and Tables

**Table 1:** The basic information about the European eels (*Anguilla anguilla*) collected from the Vistula and Oder River basins (EMUs) used in this study. Data marked with different letter indices show significant statistical differences (*p* < 0.05).

	Vistula River EMU	Oder River EMU
No. of fish	*n* = 20	*n* = 20
Fish weight (g)	265.3 ± 13.9 (184–382)	428.6 ± 47.3 (158–932)
Fish length (mm)	524.6 ± 10.2 (472–623)	580.1 ± 129.7 (447–761)
Age of fish (years)	8.8 ± 1.2 (6+ to 10+) ^a^	6.4 ± 2.1 (5+ to 13+) ^b^
Number of individuals of *Anguillicoloides crassus* (%)	76.5	70.0
Fulton factor K	0.182 ± 0.004 ^b^	0.205 ± 0.005 ^a^

## Data Availability

All data obtained in the course of this study were used to produce the results reported in this manuscript. The underlying data are not publicly available; however, they can be provided by the corresponding author upon reasonable request.
